# A Smartphone App for Adolescents With Sleep Disturbance: Development of the Sleep Ninja

**DOI:** 10.2196/mental.7614

**Published:** 2017-07-28

**Authors:** Aliza Werner-Seidler, Bridianne O'Dea, Fiona Shand, Lara Johnston, Anna Frayne, Andrea S Fogarty, Helen Christensen

**Affiliations:** ^1^ Black Dog Institute University of New South Wales Sydney Australia

**Keywords:** insomnia, sleep, adolescence, depression, cognitive behavioral therapy, smartphone

## Abstract

**Background:**

Sleep disturbances are common in young people and have consequences for academic, social, emotional, and behavioral development. The most effective treatment is cognitive behavioral therapy for insomnia (CBT-I), with evidence suggesting that it is efficacious even when delivered digitally.

**Objective:**

There are no commercially available digitally delivered CBT-I programs for use by young people. The aim of this project was to develop a smartphone app that delivers CBT-I to young people to improve sleep.

**Methods:**

To inform the development of the app, young people (N=21) aged between 12 and 16 years attended one of the 3 focus groups (each with 4-10 participants). These focus groups were conducted at different stages of the development process such that the process could be iterative. Participants were asked the reasons why they might use an app to help them sleep, the kinds of features or functions that they would like to see in such an app, and any concerns they may have in using the app. Data were analyzed using a thematic analysis approach. Of the issues discussed by the participants, the researchers selected themes associated with content, functionality, and accessibility and user experience to examine, as these were most informative for the app design process.

**Results:**

In terms of content, young people were interested in receiving information about recommended sleep guidelines and personalized information for their age group. They reported that keeping a sleep diary was acceptable, but they should be able to complete it flexibly, in their own time. They reported mixed views about the use of the phone’s accelerometer. Young people felt that the functionality of the app should include elements of game playing if they were to remain engaged with the app. Flexibility of use and personalized features were also desirable, and there were mixed views about the schedule of notifications and reminders. Participants reported that for the app to be accessible and usable, it should be from a trusted developer, have engaging aesthetics, have a layout that is easy to navigate, not rely on Internet coverage, and preferably be free. Participants felt that being able to conceal the purpose of the app from peers was an advantage and were willing to provide personal information to use the app if the purpose and use of that information was made clear. Overall, participants endorsed the use of the app for sleep problems among their age group and reported motivation to use it.

**Conclusions:**

The Sleep Ninja is a fully-automated app that delivers CBT-I to young people, incorporating the features and information that young people reported they would expect from this app. A pilot study testing the feasibility, acceptability, and efficacy of the Sleep Ninja is now underway.

## Introduction

Some researchers have suggested that sleep problems are epidemic among young people, with approximately 30% of adolescents reporting at least some level of sleep difficulty, and 10% to 40% of high school youth indicating sleep deprivation [1,2]. At the clinical end of the spectrum, insomnia is the most common sleep disorder, with prevalence rates of approximately 4% in youth [2]. Insomnia is categorized by chronic and persistent difficulty initiating sleep, maintaining sleep, or waking up too early and is associated with functional impairment [3].

Sleep disturbance experienced by young people has profound consequences across academic, social, emotional, and behavioral domains [4]. In terms of education, sleep quality, lower levels of sleepiness, and longer sleep duration are each independently associated with improved academic performance [5]. Sleep problems also have an adverse impact on behavior, with studies consistently showing that poor sleep negatively influences interpersonal relationships [6] and is associated with increased risky behaviors [7]. Poor sleep also has a marked impact on mental health generally and specifically on depression and suicidality. Longitudinal evidence has accumulated suggesting that sleep disturbance is an independent risk factor for the onset of depression [8-13]. For example, in a large community-based longitudinal study, adolescents who met clinical criteria for insomnia were between 1.5 and 3 times more likely to develop depression in the future (up to 7 years later) and up to 6.5 times more likely to attempt suicide [14]. Accordingly, sleep disturbance can seriously derail psychological, social, and vocational pathways into adulthood.

The most effective treatments for insomnia are behavioral treatments such as cognitive behavioral therapy for insomnia (CBT-I), which has consistently been found to reduce symptoms of insomnia to a greater extent than pharmacotherapy [15]. CBT-I is the first-line treatment for adult insomnia [16] and typically involves a combination of strategies, including psychoeducation, sleep restriction, stimulus control, cognitive therapy, and sleep hygiene and is usually delivered over the course of 6 to 12 weeks. A meta-analysis reported that the effect of CBT-I on sleep indices such as Sleep Onset Latency, Total Sleep Time, and Sleep Efficiency ranged from medium to large (0.67-1.09; [17]). Importantly, this approach has also been validated in adolescents [18].

Over the past decade, researchers have increasingly investigated digitally delivered CBT-I (dCBT-I) and have reported comparable effect sizes to face-to-face formats [19]. Several recent studies have directly compared dCBT-I with group and individual face-to-face formats. Results from these investigations converge on the finding that individual face-to-face CBT-I is associated with larger effect sizes than other formats, although there is no difference between group-delivered and dCBT-I, with these programs yielding conventionally large effect sizes [18,20,21]. Most of the existing research has investigated Web-based CBT-I programs, although encouragingly, a recent randomized controlled trial evaluated a fully automated CBT-I app in adults compared with a waitlist and found large effect sizes on sleep outcomes [22].

Although several digital programs are available for sleep disturbances in adults [23,24], to our knowledge, there is only one Web-based program that has been tested for use by young people [18]. This program involves 6 weeks of Web-based sessions, during which users receive individualized feedback and recommendations by a qualified sleep therapist, in addition to accessing core CBT-I components (eg, psychoeducation, stimulus control, sleep restriction, cognitive therapy, and relaxation). However, this program is not commercially available for use and requires substantial clinician input, thus limiting the accessibility of the program. The lack of automated digital programs available for use by young people is particularly surprising, given that delivering treatment digitally may have particular appeal to young people who have a preference for management of their own symptoms and problems, without the input of health professionals [25].

The aim of this study was therefore to design and develop an automated smartphone app for use by young people to improve their sleep. The scope of the app was broadened to target not only insomnia but also mild to moderate levels of sleep disturbance because insomnia is progressive and intervening early may prevent escalation to clinical levels. Using a participatory design approach, adolescents contributed their views via focus group discussions that informed the design and development process. There has been increasing recognition that involving users in the development of health information systems increases technology adoption, user satisfaction, as well as trust and ease of use of specific programs [26]. Currently, adherence rates to technology-mediated treatment are low at just 52%, suggesting that there is considerable room for improvement [27]. Using a participatory design approach may improve eventual adherence and was selected to align with best practice guidelines in the development of mental health smartphone apps [28]. According to these guidelines, understanding the needs of the end user is paramount to the eventual uptake of the app and should be systematically addressed during the development and design phases. Therefore, adoption of a participatory design aligns with these recommendations. The decision to employ focus groups was made because young people are generally comfortable and familiar with the process of discussing issues in small groups [29].

## Methods

### Design

This qualitative study involved 3 focus groups. Focus groups, rather than interviews or surveys, were selected for their specific ability to identify group norms [30], to foster interaction between participants and generate new ideas [31], as well as their capacity to encourage participation by group members who might otherwise feel that they do not have much to contribute [32]. Three focus groups, each with 4 to 10 participants, were held at different stages of the design and development process such that the process could be iterative. The first focus group was held before the commencement of the project for feedback on the concept, the second was conducted after the concept had been established, and the final group was held when a prototype was available to maximize the capacity for feedback at each stage of the design process. Participants attended only one of the 3 groups.

### Participants and Recruitment

A convenience sample of young people aged 12 to 16 years was recruited via Web-based channels, including the Black Dog Institute Web page, Facebook, and Twitter. Study flyers were also placed in community areas such as libraries and doctors’ waiting rooms. Potential participants responded to advertisements asking young people to participate in a focus group about the development of a smartphone app to improve their sleep. To be eligible to participate, respondents needed to be aged between 12 and 16 years, fluent in English, able to travel to Randwick for focus groups, own a smartphone, and have a parent willing to provide consent. Participants were not required to be experiencing current sleep disturbance because the aim of the focus groups was to elicit a wide variety of perspectives from those with significant sleep problems through to those without. However, one might assume that this study was likely to appeal to people for whom sleep is a significant concern, making it likely that the sample would include participants at the clinical end of the spectrum. This sampling approach was chosen to ensure that the app appealed to a range of young people with varying levels of sleep difficulty. Interested participants contacted the research team via phone or email to confirm eligibility and their availability to attend a face-to-face focus group at the Black Dog Institute in Randwick. Written consent forms from a parent or guardian were emailed to researchers before the focus group session. After consent had been provided, focus groups were conducted when at least four participants had signed up.

### Procedure

Focus groups were held at the Black Dog Institute and conducted by the research lead (AWS) who has a background in clinical psychology (PhD/MPsychol) and extensive experience in facilitating interviews and focus groups. No relationship between the research lead and participants was established before the focus group. A graphic designer involved in the development of the sleep app also attended the focus groups as an observer but did not contribute to the discussion. Each group session was held in a quiet meeting room and lasted approximately 90 min. The focus groups were conducted using a semistructured format, with questions generated to address areas of interest as informed by commonly identified risks and ethical considerations in app development, which include functionality, privacy issues, and usability [33]. The facilitator asked general questions about app content, before providing an overview of core content areas. Focus group participants provided their views about a range of candidate content areas such as the establishment of regular sleep-wake cycles and the introduction of a pre-bedtime routine. The questions were piloted by the research team before conducting the groups. Questions were open ended, with specific prompts being used as necessary (see Multimedia Appendix 1 for sample questions). The same question outline was used in all 3 groups. The only difference was that a prototype was presented to all the participants in the third focus group toward the end of the session, and they were therefore able to provide additional specific feedback on user experience components (eg, look and feel and age appropriateness). Focus groups were audiotaped for later transcription. Participants completed a set of 3 paper-based questionnaires (see below) at the end of the focus group discussion. They were reimbursed with a Aus $30 gift voucher for their time. All procedures received ethical approval from the University of New South Wales Human Research Ethics Committee (HC15422).

### Measures

#### Demographics

Participants were asked to provide information about age, gender, school year, main language spoken at home, and an estimate of time spent on their phone each day.

#### Insomnia Severity Index

The Insomnia Severity Index (ISI; [34]) is a 7-item self-report questionnaire based on the Diagnostic and Statistical Manual of Mental Disorders, fourth edition criterion for insomnia [35]. It assesses perceived symptoms and consequences of insomnia as well as how concerning these difficulties are. Each item is rated on a scale of 0 to 4, with total ISI scores ranging from 0 to 28. The ISI has the following cutoff points: 0 to 7 for no clinically significant symptoms, 8 to 14 for subthreshold insomnia, 15 to 21 for clinical insomnia of moderate severity, and 22 to 28 for severe clinical insomnia [36]. The ISI has been widely used among adolescent samples, with internal consistency of .83, and 2-week test-retest reliability of .79 [37].

#### Kessler 10 Psychological Distress Scale

The Kessler 10 Psychological Distress Scale (K10; [38]) is a 10-item scale that assesses levels of general psychological distress. Total K10 scores of 1 to 15 indicate low, 16 to 21 indicate moderate, 22 to 29 indicate high, and 30 to 50 indicate very high psychological distress [39]. The K10 has high internal consistency (.93) and good discriminant validity in population-based samples [40]. Additionally, the sensitivity of the K10 measure at both ends of the scale lends it to use among community, nonclinical samples [41].

### Data Analysis

Digital recordings were transcribed verbatim by a research assistant. In line with thematic analysis guidelines [42], coding was conducted by 2 primary coders (AWS and LJ) using a data-driven approach. Two independent coders reviewed the transcripts; one of the coders was already familiar with the data on account of conducting the focus groups (AWS), whereas the other listened to recordings and closely reviewed transcripts (LJ).

Thematic analysis was selected to analyze the data because of its flexibility [43] and rigor [44]. Thematic analysis is well-suited to this study because it allows for the identification, interpretation, and reporting of patterns within the data [45] while simultaneously recognizing the reflexive role of the researcher in presenting the data [46]. Data from the 3 focus groups were exported into Microsoft Excel. Both coders were experienced in conducting focus groups and interviews. A qualitative analyst (AF) oversaw the data analytic approach.

Initial coding was conducted by 2 coders on the same single transcript, each blind to each other’s codes, to generate an initial first-stage coding framework. These initial codes were discussed and refined until both coders were in agreement about the coding framework. The senior qualitative analyst then reviewed this coding framework before it was applied to the remaining 2 transcripts and reapplied to the initial transcript upon which it was based. Codes were compared between the 2 coders, and discrepancies were resolved to create detailed code descriptions that could be applied consistently to the text. Constant comparison was used to further refine the codes and generate higher-order explanatory thematic groupings in the data. This final coding framework was then applied to all 3 transcripts to test the validity and appropriateness of identified themes. The consolidated criteria for reporting qualitative research checklist was used to guide the reporting of results [47].

## Results

### Participant Characteristics

A total of 21 young people (age range: 12-16 years, mean age: 14 years; 71% (15/21) female) participated in the 3 focus groups (see Table 1 for details on participant characteristics). No participants withdrew or dropped out after the study commenced. A total of 90% (15/21) nominated English as the language spoken most at home. On average, participants estimated that they spent 3.5 hours per day on their phones (range: 30 min-9 hours). Total scores on the ISI ranged from 0 to 19, with considerable variability in severity—55% of the participants had no clinically significant symptoms, 25% of participants reported moderately severe insomnia, and 20% reported subthreshold insomnia. Scores on the K10 ranged from 11 to 37, with 33% of the participants reporting low levels of psychological distress, 33% reporting moderate levels of psychological distress, and 10% and 24% describing high and very high levels of psychological distress, respectively. Scores on the ISI and K10 were positively correlated, *r*=.76, *P*<.01.

**Table 1:** Participant characteristics.

Participants (N=21)	Mean (standard deviation)	Range
Gender (female), n (%)	15 (71)	-
Age, in years	14.10 (1.26)	12-16 years
School year	8.76 (5.65)	6-10
Language mostly spoken at home (English), n (%)	19 (90)	-
Time spent on phone per day (hours)	3.56 (2.72)	0.50-9
ISI^a^score	8.71 (5.65)	0-19
K10^b^score	20.95 (8.05)	11-37

^a^ISI: Insomnia Severity Index.

^b^K10: Kessler 10 Psychological Distress Scale.

### Thematic Analysis Results

The thematic analysis generated 3 distinct themes, which guided the development of the Sleep Ninja app. These included (1) content; (2) functionality; and (3) accessibility and user experience. An additional 5 themes were identified, which were broader in scope and related to current behavioral patterns and attitudes rather than to the development of the sleep app specifically. These included (1) current obstacles to healthy sleep; (2) current sleep strategies; (3) motivation to change sleep habits; (4) general phone usage habits; and (5) attitudes toward apps for health and sleep. Each of these themes emerged from all 3 groups, with the addition of specific feedback on aspects of the prototype presented in the focus group 3. All themes are listed in Table 2 with subcodes and illustrative quotes listed for each theme. Given the objective of the study, the detailed reporting of results and discussion will be limited to the 3 themes that directly relate to the development of the sleep app.

#### Content

Content refers to the psychoeducational material contained within the app and the information collected about users’ actual sleeping patterns.

##### Sleep Information

Adolescents expressed interest in the number of hours of sleep that are recommended for people their age. Many wanted help setting their body clock and being able to get to sleep earlier, whereas others were less receptive to the idea of being reminded to maintain consistent sleep/wake times, particularly over the weekend. There was also an interest in obtaining information about sleep in general, including why we sleep, what happens to the brain during sleep, what the stages of sleep are, and why we dream. A majority of adolescents liked the idea of including a large database of sleep tips that varied in themes ranging from relaxation strategies to factual information about the benefits of quality sleep. Participants agreed that the user should be free to enter and exit the sleep information as they wish.

##### Sleep Diary

There were mixed opinions about completing a daily sleep diary, but most adolescents thought that filling in a sleep diary was acceptable. Some adolescents preferred the idea of completing the sleep diary first thing in the morning, whereas others indicated a preference to complete the diary later in the day or as a way to fill “dead time” such as when on the bus or train to school. One issue that arose was that some young people were concerned that their recollections of sleep and wake times would not be very accurate. On the whole, adolescents were positive about having a sleep record and being able to look back on it as a way to track progress.

#### Functionality

Functionality refers to the range of operations and capabilities of the app that define the characteristics of the app.

##### Gamification

Gamification is the application of typical elements of game playing in nongaming systems to improve user experience and engagement [48]. The concept of making the app a game where the user has to “level-up” was universally endorsed. Young people liked the idea of using a belt system within the Sleep Ninja concept as a way to distinguish the different levels. The “belt system” refers to the idea that users begin with a beginner level “white belt” and progress through the levels of increasing difficulty until they become an advanced-level “black belt” in sleep. Having a series of goals that need to be attained before moving to the next level was considered a positive feature of the Sleep Ninja, and adolescents reported that it would enhance their ongoing engagement with the app:

It keeps you using the app while otherwise some people may use it for a day and then forget about it.

##### Flexibility

Participants indicated that the app should be delivered flexibly. For example, one young person suggested that the user should be able to complete the content components (training sessions) in small chunks rather than all in one go. Others suggested that being given the choice to do shortened versions of each training session as opposed to the regular length training session would add appeal:

...you could have a short training session or a long one, so there’s the option.

However, a majority of adolescents then agreed that one standard length training sessions for everyone would be superior because if there were short options they didn’t think they would be motivated to do the longer version.

##### Personalization

Several participants thought the app would be more appealing if it could be personalized. For example, one young person commented:

Sometimes when people have apps like this and it’s helping them to do with something in their life, they like it to feel like it’s connected to you so maybe something like “Hi [name]” or something related to you would be good?

Other young people felt that the app would be strengthened by having the information tailored to the user and their specific sleep issue.

##### Reminders and Notifications

Participants expressed mixed opinions about reminders and notifications. Although several young people discussed switching off notifications and ignoring reminders because they “stuff up my phone,” in the context of a sleep app, many thought reminders and notifications were acceptable:

If it were important and only up to 2 a day asking “Have you filled in your data yet?” I’d do it - it’s not annoying or anything.

A commonly expressed opinion was that reminders and notifications from a sleep app would be helpful:

If you’re getting notifications and they’re definitely going to help you...it would be good.

On the whole, young people indicated that reminders and notifications needed to be delivered at times that were suitable to the individual and that up to 2 per day would be acceptable.

##### Accelerometer

A number of participants expressed interest in using the phone’s inbuilt accelerometer to track their sleep. However, some participants were concerned about having to keep the phone under their pillow at night for a variety of reasons, which included it being uncomfortable, not being allowed to keep their phones in their room overnight, and concerns about radiation and the health implications. At least six participants did not have these concerns. Interest in the accelerometer was contingent on users being able to receive feedback from the app about their sleep.

##### Extra Features

Adolescents provided a “wish list” of features they would like to see included in the sleep app. These included calming music/and or stories to read; a meditation recording; an inbuilt function that disables communication at a certain time to remove ability to use social media; a hotline or Web-based portal to discuss sleep problems and strategies with an expert; personal stories of sleep strategies that have worked for other people; an option to have the text read aloud to you; a chatroom to share advice with other users; tangible rewards for improving sleep habits; and a dream diary/notes section.

#### Accessibility and User Experience

As suggested by the code label, acceptability and user experience relates to attitudes, emotions, and experience of users interacting with the app.

##### Trust

Participants indicated that trusting the source of an app before downloading it was important. Whereas most participants endorsed the Black Dog Institute to be a trustworthy organization, there was the perception that same-aged peers would be unlikely to recognize the brand. Young people agreed that they often relied on the look of the logo to make decisions about how trustworthy the app is (“It just has to have a good logo.”). Developers who have made more than a single app or that have user reviews were considered more trustworthy. Participants did not report evaluating the credentials of the developer to assess trust issues nor did they raise scientific quality or validity as important factors in determining trust.

**Table 2:** Themes, subthemes, and illustrative quotes for each theme that emerged in the thematic analysis.

Theme	Subtheme	Examples
Content	Sleep information	“A sleep calculator. So, depending on age and things, when you should go to bed or when you should wake up.” “It would have lots of different information and ideas and strategies.” “It could have fun sort of facts like if it says you get this amount of sleep, it improves your actual mood, your awareness, your reflexes.”
	Sleep diary	“I would do it 5 times a week it’s just at the weekend because you don’t really know when you wake up.” “...cause with the tracking, I might not do it as soon as I wake up but maybe once I leave the house when I’m like on the school bus.”
Functionality	Gamification	“I think it’s good that you can unlock levels...it encourages you to use the app.” “That’s good for young people as young people want to play games.”
	Flexibility	“You could have a short training session or a long one.” “I’m not that keen on the long and short version idea, I think it would be better to say, there are this many questions in the session and it will take approximately this amount of time and when you have the time you sit down and do that.”
	Personalization	“...different types of information for people with different sleeping problems - something like “Hi [name]” or something related to you.”
	Reminders and notifications	“If it were important...asking “Have you filled in your data yet?” I’d do it, it’s not annoying.” “An option that we can swipe across and snooze it so that you can set it again later.” “Notifications I just ignore in general.”
	Accelerometer	“A lot of parents make you sleep with your phones outside of your bedrooms so they wouldn’t want you to sleep with your phone under your pillow.” “I would actually be really interested...so you can tell what helped you sleep.”
	Extra features	“A social media thing, so you could...post a message asking for help.” “...kind of like Siri, so you would ask her like, questions about your sleeping, and she would give you a list of options or something.”
Accessibility and user experience	Trust	“There’s a lot of dodgy app makers but if it’s some trustworthy - like you guys [Black Dog Institute], then I would [trust it].” “The logo is really important.”
	Privacy	“I feel secure, I like the password.” “I think the “Sleep Ninja” is really good because you can say it’s a game.”
	Data security and confidentiality	“I usually put in fake details.” “Sometimes I don’t like, trust the app. Coz I could do my own thing and they could steal my identity, for all I know.”
	Data usage and Internet coverage	“If I’m gonna download an app that requires a lot (of data) I’ll wait awhile.” “I don’t really use Internet that much. I restrict my apps.”
	Cost	“Okay um 50/50 an app costing something is not necessarily going to turn you off.” “I’m just happy to tolerate some ads and stay with the free version.”
	Age-appropriateness	“I think it’s good for people our age group...it’s very simple to use.”
	Look and feel	“It just has to have a good logo.” “Cartoons are good because they’re just kind of easy to recognize.” “...it needs to have a good layout and easy to navigate.”
	Predicted likelihood of using Sleep Ninja	“I would definitely download it and I would do it for a couple of days but I don’t know if I’d get sick of it.”
Obstacles to sleep	Technology use	“When you’re using Instagram or Facebook you’re kinda going quickly so if you’re in that mindset before going to sleep you can’t really relax completely.”
	Cognitive and emotional factors	“Sometimes I overthink and then I can’t get to sleep because I’m overthinking.” “...if I’m not getting to sleep, I start stressing that I can’t get to sleep because I worry that the next day I’m going to be tired and I keep on thinking about things and I worry.”
	Environmental factors	“...even if the door is slightly open and there is a little bit of light coming in, it’s also disrupting. And if I’m sleeping in a spot that’s not my usual bed.”
	School commitments	“...with group assignments we often have to do all-nighters and things.”
General phone habits		“I always carry my phone with me.” “I would feel like half empty [if I forgot my phone]. Coz, I would be thinking the whole time like, has someone messaged me?” “I’m not allowed to sleep with my phone in my room but I hide it.”
Bedtime habits		“I sometimes put my phone on aeroplane mode right before I go to bed.” “I usually do something that doesn’t help me go to sleep. I will go through my Twitter timeline and my YouTube feed...does keep me awake for a long time.”
Attitudes toward apps for health		“If there was something you had a problem with, you would want to go see someone but if it was something you were more curious about...like, I wonder if doing this affects me, you would get an app.” “I reckon if I did have a sleeping problem, I would be interested.”
Motivation for changing sleep habits		“I would do it, if, in the long term it would affect me in a positive way.” “I sleep well, well...not well, but I don’t really care about how I sleep, like I’m not a really bad sleeper but I don’t really mind.”

##### Privacy

Participants discussed the idea of concealing the true purpose of the app from their peers. They indicated that a password or lock on the app would be a positive feature. A suggested strategy to enhance privacy was to use an innocuous name for the app:

...with the design for the app, someone said something earlier about feeling self-conscious about it so I think [calling it] the “Sleep Ninja” is really good because you can say it’s a game.

##### Data Security and Confidentiality

Participants raised general confidentiality concerns that they have with apps. Willingness to provide personal information was largely contingent on whether they could understand the reason for needing to sign in and/or provide personal information. Participants did not raise any issues or concerns with data security or storage.

##### Data Usage and Internet Coverage

Participants discussed different phone data plans and reported a range of levels of concern with the data usage from no concern (“I’ve got heaps of data.”) to a lot of concern (“You don’t wanna use your 3G.”). Many participants reported downloading apps that run without Internet coverage.

##### Cost

Some participants had previously paid for apps, whereas others would rather tolerate ads and use only apps that are free. There were mixed opinions on whether they would pay for apps, and those who would, mainly said that they would pay for games or Spotify, and some indicated it would be their parents who would pay for an app.

##### Age-Appropriateness

The second and third focus groups were shown images of the app, which were rated favorably (“They’re easy to understand, they’re pretty clear.”). Participants generally felt that the images were age-appropriate and felt that the gamified component (see System Design section below) of the app was considered particularly relevant to their age group.

##### Look and Feel

The aesthetic aspect of the app generated a lot of discussion and was important to all participants. Common preferences were that the app had a simple logo and short name and that these factors contributed toward the impression that the app would be of a good quality. The Sleep Ninja images were generally viewed positively, however, some participants saw them as being too detailed and felt that these could be improved by minimizing the shading and relying just on outlines and block colors.

##### Predicted Likelihood of Using Sleep Ninja

There were mixed ratings of likelihood to use the Sleep Ninja, ranging from 2 to 9 out of a possible 10. Some participants expressed disinterest because of healthy sleep habits already or not prioritizing changes to their sleep patterns. Other good sleepers did not agree and indicated that they would use it. Whereas most participants thought that they were more likely than not to download the app, the motivation to continue using the app was more commonly questioned:

I would definitely download it and I would do it for a couple of days but I don’t know if I’d get sick of it

### System Design

The information raised in the focus group discussions informed a number of key changes made in the Sleep Ninja app:

#### Content

Specific sleep information requested by young people (eg, sleep stages, reasons for sleeping, and what happens to the brain during sleep) was built into the app in an “Information Section.” A database of “Sleep Tips” was also incorporated into the app. As requested by young people, both of these content areas were optional and could be entered and exited at any time by the user. With respect to the sleep diary, the app now includes prompts for the user to complete it first thing in the morning, because young people indicated that they may forget. However, in line with the importance of flexibility expressed by participants, the prompt can be dismissed, and users can complete the diary at any time of the day. Users are also able to access their own past sleep diary data as a way to monitor their progress.

#### Functionality

The positive consensus about the gamification of the app led to the confirmation of the belt system concept, by which participants could “level up.” To align with young people’s preferences in terms of flexibility and consistency, the app comprises standard lessons that do not vary in length from user to user, but users can complete as little or as much of each lesson as they wish. Upon reentering the app, the users can then pick up exactly where they left off. Although the app is not personalized to the user in terms of specific details such as their name, the chat-bot structure allows it to be responsive to the needs and interests of the user by asking questions about what they would like to know more about and what issues they are experiencing, and it can also tailor recommendations about sleep times and efficiency goals based on the data inputted by the user. Focus group participants felt that reminders and notifications would be helpful but had to be delivered at suitable times and should not be too frequent. Therefore, notifications are delivered first thing in the morning when the user has indicated they would like to get up and 1 hour before the recommended bedtime. These notifications are configurable and can be “snoozed” should the user be unable to respond immediately. An option to turn off notifications at any time was also built into the design. The accelerometer function has been added as an optional feature; however, given the concerns expressed by young people about keeping their phone close to them while they sleep, this is not a core component of the app. Finally, most of the extra features suggested by young people were beyond the scope of the app because they either did not accord with the best practice guidelines for the treatment of sleep problems (eg, a dream diary), they were impractical (eg, tangible rewards for implementing sleep strategies), or contradicted privacy issues that young people brought up (eg, sharing stories in a chatroom of users). However, in response to requests from young people about a strategy they could use while trying to fall asleep, the inclusion of a meditation recording was incorporated into the app. Although not a core part of all CBT-I programs, the use of relaxation and meditation strategies have been incorporated into some standardized programs (eg, [18]). The need for young people to be able to reach an expert led to the inclusion of a “Get Help Now” function with a direct link to an existing Australian 24-hour crisis helpline.

#### Accessibility and User Experience

Some participants expressed that trusting the source of an app was important, so to address this, information about the app developers with a link to the organization’s website was included in the “Frequently Asked Questions” section. The app was designed to look like a game, based on participant feedback regarding privacy and discretion. The potential for a passcode has been built in, but given that no sensitive personal data are stored in the app, the use of this feature will be determined by user feedback at a later stage. Similarly, given that no sensitive data are collected and stored by the app, there is no need for users to provide personal information, which was a concern for young people. Participants reported a preference for apps that do not require a significant amount of data or continuous Internet coverage. Therefore, the download and use of the Sleep Ninja app is not contingent on an Internet connection, with data uploads to occur only when the device is connected to a Wi-Fi network. There will be no cost to access the app while it undergoes testing. Should it be effective, every effort will be made to keep the app free, but this will depend on the continued cost of making it publicly available. No changes were made to the content to make it more age-appropriate, as the participants agreed that it was suitable for their age-group. The look and feel of the app was simplified based on focus group discussions, with unnecessary detail being removed.

**Figure 1:**
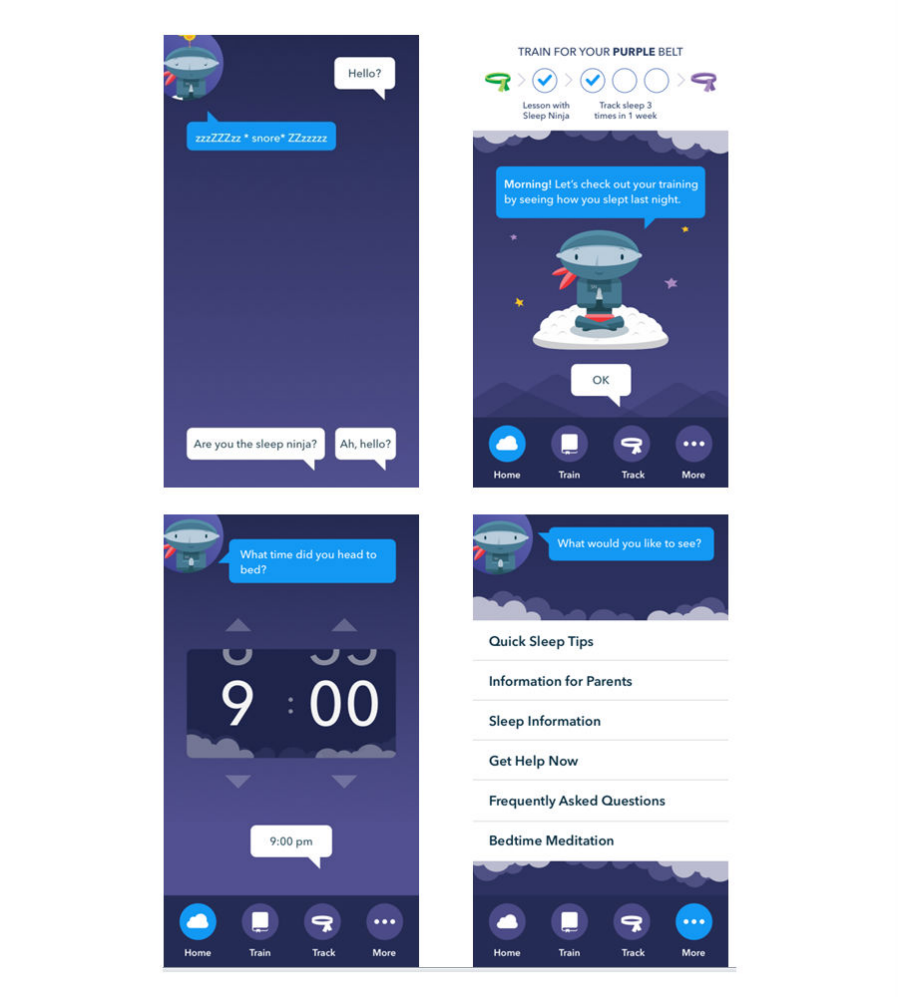
Top-left panel shows Sleep Ninja chat-bot format for training sessions with forced choice options for user at the bottom of the screen; Top-right panel shows home screen where the user can choose to train or track their sleep. An index of what the user needs to do to “level-up” to the next belt is shown at the top of the screen; Bottom-left panel shows an example of completing the sleep diary; Bottom-right panel shows options in the “More” section.

Combining this information garnered from the focus groups, with several core strategies of CBT-I, the Sleep Ninja was created. The structure of the app includes 6 training sessions (lessons), a sleep tracking function, recommended bedtimes based on sleep guidelines, reminders to start the wind-down routine each night, a series of sleep tips, and general information about sleep. The home screen has 3 options: Train, Track, or More (see Figure 1). The Train tab is where users complete the training sessions, each of which covers a core strategy that is delivered through a chat-bot format where the sleep ninja essentially acts as a sleep coach. The core strategies include psychoeducation (training 1), stimulus control (training 2), daytime behavioral activation to improve tiredness at night and sleep hygiene (training 3), identifying and planning for high-risk situations (training 4), cognitive therapy (training 5), and a final review session (training 6). As requested by the participants, the user interacts with the app through the forced choice chat-bot format, so information can be personalized to what they want to know about, without the need to type. Users level up and reach their next “belt” by completing one training session and tracking their sleep for 3 nights (out of a 7-night period).

The Track option allows the user to enter data about how they slept last night, and set a wake-up time for tomorrow. This provides the information necessary for the ninja to prompt the user to start their wind-down period an hour before bedtime. Users receive a report at each level about their progress, and when they complete all 6 levels, they become a “black belt” in sleep. The More section contains over 40 different sleep tips, general sleep information and frequently asked questions, details of crisis helplines, a link to email parents information about the app, and a bedtime relaxation exercise. The app is not designed to be used at night, but a relaxation recording was included based on what young people wanted, and so that there was something practical they could use in the moment should they be trying to fall asleep.

## Discussion

### Principal Findings

This study outlines the involvement of young people in the development and design of a smartphone app, the Sleep Ninja, which is a CBT-I based program to help adolescents with their sleep. The adolescents who participated in this study declared a strong interest in the app concept, and most reported that they would be willing to use the app if experiencing poor sleep. However, the likelihood of participants’ use of the app was found to be influenced by the sleep content included in the app, its functionality, and user-experience characteristics. Participants made a number of recommendations for how the app could be improved, which included a section outlining the scientific basis for the need to sleep, the ability to enter and exit the app flexibly, tailoring of information to meet the needs of the specific user, limiting reminders to 2 per day, adding a “Get Help Now” function, and the simplification of the app layout and color palette. Participants who saw the prototype were able to provide specific guidance regarding the look and feel including the colour scheme. Most notably, across the focus groups, the young people indicated a preference for gamification, which is not surprising, given the adoption of gaming strategies in other health apps (eg, [49,50]) and the general popularity of games in this age group [51]. These recommendations were accepted in that the app is now delivered in a gamified format with a leveling-up component.

The sample of young people who participated in this study was spaced across the full spectrum of insomnia and psychological distress, with scores from normative levels to the clinical range. This suggests that multiple viewpoints and perspectives in terms of sleep difficulty and psychological distress were provided in the focus group process. The finding that 45% of participants reported at least some clinically notable level of sleep disturbance is commensurate with prevalence estimates in this age group [1,2], as were estimates of psychological distress [52]. The significant correlation between sleep disturbance and psychological functioning in this sample provides evidence of the link between poor sleep and adverse psychological outcomes. The variability in scores and comparability to prevalence estimates confirm that this was a representative and appropriate sample to consult with for the development and design of the Sleep Ninja app.

Even though CBT-I is the gold standard treatment for sleep difficulties, it is not always easily accessible to young people. Adolescents are unlikely to seek professional help for their sleep problems and report a preference for self-reliance methods [53]. The introduction of a smartphone app for the management of sleep disturbance is in line with the preferences of young people, as it is an automated, self-directed tool. A potential concern associated with a low-intensity fully automated app is that some users may require additional support but may not follow up with support services. This aligns with previous work indicating that clinician support for the use of the app can be helpful, particularly in clinical groups [54]. However in this study, users are continually provided feedback while using the app and encouraged to seek help from their doctor or a trusted adult if their symptoms do not improve. This advice, coupled with the easy access to crisis helpline numbers embedded in the app, encourages help-seeking behavior among users who require additional support.

The context in which the app will be delivered and adopted by users will require careful consideration. For example, participants indicated that they seldom search for things off the app store and are likely to get suggestions from friends and families. One possibility is to introduce the app within the school setting such that teachers and school counselors can recommend it for use to young people and their families to treat sleep disturbance, with the possibility of ongoing monitoring. This will also help to mitigate any risks associated with young people failing to seek additional help when they need to. However, before this issue is addressed, the next step in this phase of work is to empirically test the feasibility, acceptability, and efficacy of the app. A pilot trial to address these issues is now underway in Australia (#ACTRN12617000141347).

### Limitations

Several limitations to this study warrant mention. First, focus group participants ranged across the continuum in terms of their level of sleep disturbance. Although this had the advantage of incorporating a range of opinions because of the diversity in the sample, it is unclear whether some groups (eg, those with clinical levels of insomnia) are more likely to respond better than others to the app or would perceive certain app features as more crucial than others. This will need to be verified in the next phase of testing. Moreover, the sample size of the study was relatively limited, and focus group methodology is associated with bias in terms of those who volunteer to participate. Although both these factors potentially limit the generalizability of the findings, diversity in the sample in terms of how they were recruited and their level of sleep disturbance in part mitigate this issue. The second limitation is that the focus groups were conducted across different phases of the development process in order to be iterative. This meant that there were fewer participants who contributed to each specific stage of development. However, the semistructured approach taken to conduct the focus groups elicited participants’ own views and opinions in general, before specific features or functions associated with the app were presented. As the amount of detail presented about the app was a function of the development stage, the groups did not respond to exactly the same material. Although this is a limitation of the study, it nonetheless allowed for feedback at different points of development, which was deemed important so that the process could be iterative.

Key strengths of this study include the participatory design approach, the automated nature of the intervention, the ability to use the Sleep Ninja on both the iPhone and Android operating systems, and the fact that the Sleep Ninja can be readily scaled to reach large numbers of young people. Although the app has been developed in Australia, there is no reason why it is not suitable for broader use (although it would require crisis helpline information to be adapted).

### Conclusions

Sleep disturbance during adolescence at both a subthreshold and clinical level has tremendous impact on broad areas of functioning across social, educational, behavioral, and emotional domains. With up to 40% of young people affected by sleep issues [1,2], establishing healthy sleep patterns early in life among this group has great potential to change a lifelong trajectory toward poorer academic performance, behavioral problems, and the onset of mental illness. Information from both the broader literature and the focus groups we report here specifically suggests that not only is there a need for such an app but also that young people are willing and open to adopt its use. Although several digital programs based on CBT-I exist (eg, SHUTi and Sleepio), these have not been designed specifically with young people in mind. Such programs tend to be text-heavy, include content that may not be suitable for adolescents, and can be expensive, making them less appealing and inappropriate for a younger age group. Incorporating elements that have been tailored specifically on the expressed needs of young people, as is the case for Sleep Ninja, is more likely to keep them engaged with the program and will be more suitable than existing adult programs.

## Appendix

Multimedia Appendix 1 Focus group sample questions.

## Abbreviations

CBT-I: cognitive behavioral therapy for insomnia
dCBT-I: digitally delivered cognitive behavioral therapy for insomnia
ISI: Insomnia Severity Index
K10: Kessler Psychological Distress Scale
