# Supplementary material for: A Smartphone App for Adolescents With Sleep Disturbance: Development of the Sleep Ninja
Source: JMIR Ment Health. 2017 Jul 28;4(3):e28. doi: 10.2196/mental.7614 (PMC5554352; doi:10.2196/mental.7614)
Supplement: Multimedia Appendix 1 [file mental_v4i3e28_app1.pdf]

1. General app use

What Apps do you use that you really like? What do you like about those Apps? How often do you use these Apps?

- What is your favourite app?
- What incentives would make you want to use an app?
- How often do you download/use apps?
- How do you decide which apps to download?
    a. Price? Do you have a budget?
    b. Word of mouth – Do your friends recommend apps?
    c. Store recommendations and reviews?
    d. Internet access requirements?
    e. Do you care about the developer or the organisation behind the app?
    f. Do you ask for parental permission?
    g. Do you care about privacy features? How do you assess this? Do you like to have an app password protected or is your phone passcode enough?
- When do you decide to stop using an app? Do you ever delete apps? Why? How long after?
    a. Is it because of annoying features like ads or that you have forgotten your log in?
    b. Does information become irrelevant?
    c. Do you lose interest?
    d. Does it have a particular purpose?
    e. Any other reasons why you would stop using an app?
- Can you think of an app that you didn't like? Why didn't you like it?
- What are some of the annoying things you experience in using apps?
    a. Ask about passwords and PIN numbers
    b. What will make it less likely to return to an App

2. Sleep

What do you think about the idea of using an App to help young people improve their sleep?

a. What would be good about it?
b. What would be not so good about it?
c. Would people need to have sleep problems in the first place?
d. Would you be interested in using an App that was designed to make you sleep better?

What strategies do you think would help young people with their sleep?

a. What strategies have you found helpful in the past?
b. What advice would you have for a friend who was having trouble sleeping?

Provide overview of app concept and content, and demo screens (when relevant)

3. **General feedback on concept**
    - What do you understand about the app based on that explanation?
    - What doesn't make sense? Do you get it?
    - What appeals? What doesn't?
    - What do you think of this? What do you like/dislike?
    - What would you look for in something like this?
    - Would you ever use it? Why/why not?
    - What features would you want in such an app?
    - How would you feel if you had to put your phone under your pillow to track your sleep
    - Are you interested in sleep data (e.g., via fitbits, jawbones etc)?
    - What about an app that asked you to go to sleep later to improve your sleep efficiency?
    - What if an app instructed you to get up earlier on the weekends?

4. **Content**

    - Which content area are you most interested in?
    - Which content area are you least interested in?
    - Would you be motivated to work through all of the lessons?
    - What do you think about not letting users progress to the lesson unless they track their sleep and complete the content?
    - Is there any other information about your sleep patterns/advice you would want the app to give you?

5. **Tracking**
    - When is the best time to ask about last nights' sleep?
    - What time do you get up?
    - What time do you leave your house?
    - How long does your sleep inertia (grogginess) usually last?
    - What time would you be most likely to fill in a sleep diary?
    - What can we do to make it more likely that young people will do this?
    - What time do you get home after school?
    - We plan to use notifications to remind people to complete their sleep tracking. How do you generally responds to alerts on your phone? Do you ignore/pay attention?
    - Do you have any tips about what would increase the likelihood of paying attention to alerts for this app?

6. **Suggestions to improve the app and branding**
    - Do you think there will be differences between the way boys and girls use this app?
    - What age do you think the app is suitable for?
    - Are there any changes you would make to the app?

- What do you think the logo should look like?
- How do you work out if an app is trustworthy?
- Should we make it clear it’s a Black Dog Institute app?

How likely would you be to use an app like this?

How likely are you to recommend this to a friend?
